# Donor-But Not Recipient-Derived Cells Produce Collagen-1 in Chronically Rejected Cardiac Allografts

**DOI:** 10.3389/fimmu.2021.816509

**Published:** 2022-01-19

**Authors:** Saidou Balam, Simone Buchtler, Frederike Winter, Kathrin Schmidbauer, Sophia Neumayer, Yvonne Talke, Kerstin Renner, Edward K. Geissler, Matthias Mack

**Affiliations:** ^1^ Department of Nephrology, University Hospital Regensburg, Regensburg, Germany; ^2^ Regensburg Center for Interventional Immunology (RCI), University of Regensburg, Regensburg, Germany; ^3^ Department of Surgery, University Hospital Regensburg, Regensburg, Germany

**Keywords:** transplantation, fibrocytes, collagen-1, allograft fibrosis, chronic rejection

## Abstract

Fibrosis is a prominent feature of chronic allograft rejection, caused by an excessive production of matrix proteins, including collagen-1. Several cell types produce collagen-1, including mesenchymal fibroblasts and cells of hematopoietic origin. Here, we sought to determine whether tissue-resident donor-derived cells or allograft-infiltrating recipient-derived cells are responsible for allograft fibrosis, and whether hematopoietic cells contribute to collagen production. A fully MHC-mismatched mouse heterotopic heart transplantation model was used, with transient depletion of CD4^+^ T cells to prevent acute rejection. Collagen-1 was selectively knocked out in recipients or donors. In addition, collagen-1 was specifically deleted in hematopoietic cells. Tissue-resident macrophages were depleted using anti-CSF1R antibody. Allograft fibrosis and inflammation were quantified 20 days post-transplantation. Selective collagen-1 knock-out in recipients or donors showed that tissue-resident cells from donor hearts, but not infiltrating recipient-derived cells, are responsible for production of collagen-1 in allografts. Cell-type-specific knock-out experiments showed that hematopoietic tissue-resident cells in donor hearts substantially contributed to graft fibrosis. Tissue resident macrophages, however, were not responsible for collagen-production, as their deletion worsened allograft fibrosis. Donor-derived cells including those of hematopoietic origin determine allograft fibrosis, making them attractive targets for organ preconditioning to improve long-term transplantation outcomes.

## Introduction

Fibrosis is characterized by excessive production of extracellular matrix (ECM) and a major cause of chronic loss of solid graft function (1–4). Several sources of matrix producing cells have been described in various models of organ fibrosis. These include fibroblasts, epithelial cells, endothelial cells, vascular smooth muscle cells, pericytes and cells of hematopoietic origin. Differentiation and activation signals are required to induce matrix production in these cells. Their contribution to matrix production is not fully understood and depends on how fibrosis is induced (type of disease) and which organs are affected. In addition, technical limitations also impede clear identification of matrix-producing cells, as e.g. fate tracking and imaging depends on specific markers or genes to identify various cell types. It is also relevant which matrix proteins (collagens, proteoglycans or glycoporteins) are used to define a matrix-producing cell. Single-cell RNA sequencing has brought new insights into the cellular origin of matrix-producing cells, however, this method preferentially detects cells with high matrix protein expression (5). As fibrosis is a chronic and slow process, cells with low matrix protein expression could significantly contribute to fibrosis, especially if they outnumber those with higher expression levels. In a highly inflammatory model of renal fibrosis (unilateral ureteral obstruction and adenine nephropathy), we previously found that hematopoietic cells contribute 30-50% to the production of collagen-1 (col1a1); the contribution of hematopoietic cells was more pronounced at later stages of fibrosis (6).

Allograft fibrosis is a prominent feature of chronic rejection that contributes to abnormal stiffness and function of the myocardium. Since chronic rejection is considered a major cause for allograft loss several years after transplantation, it will be critical to understand which cell type and derivation (donor or recipient) contribute to allograft fibrosis. Previous studies using sex-mismatched heart transplantation (female to male recipients) and lineage tracing by detection of male cells in female allografts have produced conflicting results whereby both donor- and recipient-derived cells may contribute to matrix production (7–9). To clarify this question, we used a mouse heart transplantation model in combination with selective knock-out of collagen-1 (donor or recipient) to determine the true origin of collagen-1 in organ allografts. To achieve myocardial fibrosis approximately 3 weeks after heart transplantation, CD4^+^ T cells were transiently depleted to prevent acute rejection in fully MHC-mismatched mice.

We found that donor-derived cells are fully responsible for collagen-1 production, whereas recipient-derived cells do not produce collagen-1 in the allografts. Furthermore, two different strains of mice with a cell-type specific deletion of collagen-1 in hematopoietic cells were used to determine their basic lineage. Our results show that donor-derived hematopoietic cells contribute directly to collagen-1 production in allografts. More in-depth experiments revealed that M-CSF-dependent tissue resident macrophages were not involved in this collagen-1 production, suggesting the involvement of another hematopoietic cell type.

## Materials and Methods

### Mice

14-16 week old wild-type C57BL/6 (B6) mice and 8-10-week old wild-type BALB/c (Bc) mice were purchased from The Jackson Laboratory (Bar Habor, MA, USA). The col1a1^fl/fl^, constitutive ubiquitous Cre-expressing transgenic Ubi-Cre (UbiCre), CD45^wt/cre^ and Vav1-Cre [B6.Cg-Tg(Vav1-cre)A2Kio/J, (VavCre)] mice on a C57BL/6 background were obtained as described previously (6). VavCre mice were obtained from The Jackson Laboratory. The *CD45^wt/cre^* knock-in mice on a C57BL/6 background were provided by A. Medvinsky (University of Edinburgh, Edinburgh, UK). C57BL/6 mice with constitutive ubiquitous expression of Cre by a cytomegalovirus promoter (UbiCre) were obtained from Genoway (Lyon, France). All mice were bred and housed under specific pathogen-free conditions in the animal facility, at the University Hospital of Regensburg (Regensburg, Germany). Food and water were provided ad libitum.

### *In Vivo* Macrophage Depletion

BALB/c mice (as donors) were treated with rat anti-mouse CSF1R antibody (clone AFS98, BioXCell) or rat IgG2a isotype control (BioXCell) (West Lebanon, NH) to deplete tissue-resident macrophages (10). Here, mice were treated intraperitoneally (i.p.) 3 x weekly for 3 weeks with 200 μg of either anti-CSF1R antibody or rat IgG2a isotype control antibody.

### Heart Transplantation

Heterotopic transplantation of hearts was performed as previously described (11, 12). Briefly, donor hearts were perfused twice *via* the abdominal vena cava and the pulmonary artery with cold 0.9% saline containing 500 IU heparin (3ml each; Ratiopharm, Ulm, Germany). To deplete CD4^+^ T cells, recipients were treated on day -1, 0 and 7 with i.p. injections of 1 mg of αCD4 antibody (Clone: GK1.5; BioXCell, West Lebanon, NH). We have shown previously that injection of GK1.5 completely depletes CD4^+^ T cells from the peripheral blood, spleen and transplanted heart until day 20 (13).

### Histological Analysis of Allograft Sections

As described previously (12, 13) sections of the tissues were harvested from the allografts on day 20 and embedded in paraffin or cryopreserved in Tissue-Tek compound (Sakura Finetek Germany GmbH, Staufen, Germany). Masson’s trichrome staining was performed on paraffin-embedded sections (2-3 µm) according to the manufacturer’s protocol (Sigma, Munich, Germany). For immunohistochemical staining of CD3^+^ and MAC-2+ cells, paraffin sections (2-3 µm) were incubated overnight at 4°C with anti-human CD3 antibody cross-reactive with mouse CD3 (Clone: CD3-12; Bio-Rad Laboratories, Inc., Germany) and anti-mouse/human MAC-2 (Galectin-3) (clone: M3/38, Cederlane, Burlington, Canada), respectively. After washing with PBS, slides were incubated with secondary biotinylated goat anti-rat antibody (Santa Cruz, Heidelberg, Germany) and with SensiTek-HRP (ScyTec Laboratories, Logan, Utah). The DAB-Kit (3, 3’-diamino-benzidine-tetrahydrochlorhydrate; Merck, Darmstadt, Germany) was used to visualize positive signals. For immunofluorescence staining, cryosections (3 µm) were fixed with ice-cold acetone, blocked with superblock blocking buffer (Thermo Fischer Scientific), and incubated for 1h with different primary antibodies against collagen-1 (ab21286);, αSMA (clone: E184, ab32575), both from abcam (Cambridge, UK), and fibronectin-1 (clone: AV41490; Sigma). Secondary Alexa Fluor 594-labelled F(ab)_2_ fragments of goat anti-rabbit IgG (A-11072; Invitrogen, Carlsbad, CA) and goat anti-rat IgG (A-11007; Invitrogen) were used for detection. DNA was labelled with Hoechst 33342 (Invitrogen). Images were taken with an Axio-Observer-Z1 microscope (Carl Zeiss, Oberkochen, Germany). For automated analysis, at least 10 high-power fields (HPF) were selected per slide and analyzed with MetaMorph software (Version 4.6, Universal Imaging Corp., Dowingtown, PA). Analysis was performed in a blinded fashion (12, 13).

### RNA Isolation and Real-Time PCR

RNA was isolated on day 20 from cardiac grafts after tissue homogenization in 800 µl TRI reagent (Sigma, Munich, Germany), according to the manufacturer’s instructions. Briefly, total RNA (4 µg) was reversely transcribed using Oligo (dT)_20_ primers and M-MLV reverse transcriptase (Life Technologies, Carlsbad, CA). Quantitative real-time PCR was performed using the QuantiTect SYBR Green PCR Kit (Qiagen GmbH, Hilden, Germany) and the ViiA7 detection system (Life Technologies, Carlsbad, CA). Detailed primer information is provided in the **Supplementary Table**. Relative gene expression was determined using the 2^-Δ;CT^ method (normalized to the geometric mean of the expression levels for the housekeeper *hprt1, β-microglobulin and gapdh*) (12, 13).

### Flow Cytometry

As described previously (12, 13) graft tissue was cut into small pieces and single-cell suspensions were obtained using cell strainers of 70 µm, followed by 30 µm strainers (Miltenyi Biotec GmbH, Bergisch Gladbach, Germany). Cells were then stained with conjugated antibodies against CD45 (clone 30-F11; Brilliant Violet), CD11b (clone M1/70; Pacific blue), F4/80 (clone BM8; Allophycocyanin), MHC class II (clone M5/114.15.2; APC-Cy7), CD19 (clone 1D3; PerCP5.5), CD8 (clone 53-6.7; PE-Cy7), all from BioLegend (San Diego, CA) and CCR2 (clone MC-21; PE) (14). Counting beads (Invitrogen, Carlsbad, CA) were added to each sample tube. FACS Canto II flow cytometer (BD, Heidelberg, Germany) and FlowJo (Tree Star Inc., Oregon, and USA), as well as FACS DIVA software (BD) were used for acquisition and cell analysis.

### Statistics

All data, unless otherwise specified, are shown as the mean ± standard error of the mean (SEM) and were compared using a Mann-Whitney U test. Graph Pad Prism software, version 8.0.1 was used for analysis.

## Results

### Collagen-I - Deficient Mice as Recipients and Donors of Cardiac Allografts

Conditional col1a1-deficient mice on a C57BL/6 background were described previously and contain two LoxP sites flanking exons 47 and 51 of the *col1a1* gene (6). Briefly, homozygous col1a1^fl/fl^ mice were crossed with constitutive ubiquitous Cre-expressing transgenic C57BL/6 mice (Ubi-Cre) and further sub-crossed to obtain heterozygous collagen-1a1-deficient mice (UbiCre.col^wt/fl^) and wild-type control mice (UbiCre.col^wt/wt^). Due to embryonic lethality no homozygous collagen-1-deficient mice (Ubi-Cre^+^.col^fl/fl^) were born (6, 15). Mice with a complete deficiency of collagen-1 in hematopoietic cells were generated by crossing C57BL/6 col1a1^fl/fl^ mice with C57BL/6 CD45^wt/cre^ knock-in mice or with Vav1-Cre transgenic mice. Sub-crossing resulted in CD45^wt/cre^.col^fl/fl^ mice and the controls (CD45^wt/cre^.col^wt/wt^ and CD45^wt/wt^.col^fl/fl^), or VavCre.col^fl/fl^ mice and the controls (VavCre.col^wt/wt^ and col^fl/fl^). Collagen-1 deficient mice and controls were used as donors or recipients of cardiac allografts as summarized in **Supplementary Figure 1**. Heterotopic transplantation was performed in a fully MHC mismatched setting (Bc→B6 background or vice versa). Acute rejection was prevented by CD4^+^ T cell depletion resulting in a chronic rejection with pronounced fibrosis (11–13).

### Donor-Derived Resident Cells are the Main Producers of Collagen 1 in Cardiac Allografts

**Figure 1:**
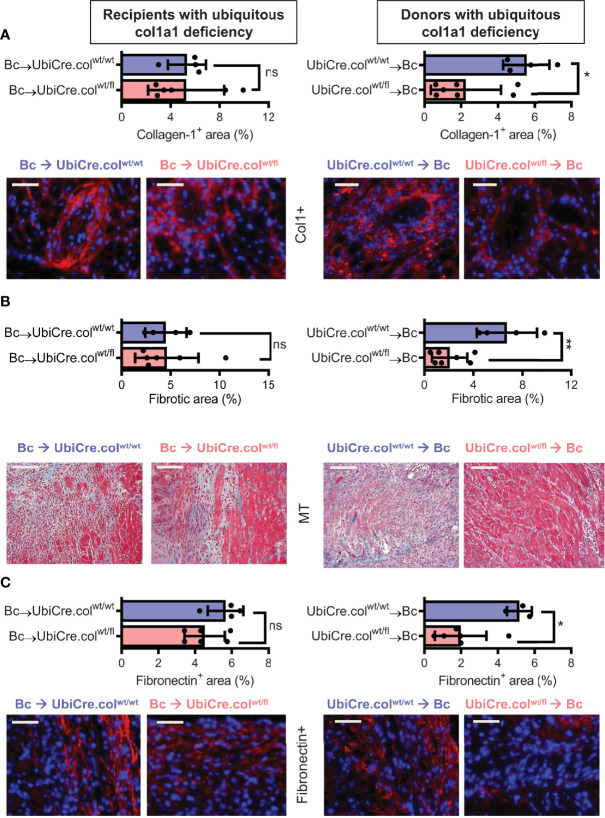
Mice with a heterozygous ubiquitous deficiency of collagen-1 used as recipients or donors of cardiac allografts - Quantification of fibrosis. C57BL/6 mice with a heterozygous ubiquitous deficiency of collagen-1 (Ubicre.col^wt/fl^) or the appropriate control (UbiCre.col^wt/wt^) were used either as recipients or donors of cardiac allografts. Recipients were depleted of CD4^+^ T cells and allografts were analyzed on day 20-post heart transplantation (HTx). **(A)** Collagen-1 positive (collagen-1^+^, col1^+^) area (%) and representative images (collagen-1 in red). **(B)** Fibrotic area (%) and representative Masson–Trichrome (MT) stainings (fibrosis in blue). **(C)** Fibronectin positive (Fibronectin^+^) area (%) and representative images (fibronectin in red). In each of the two donor groups one sample is missing because not enough tissue was left for fibronectin detection. Scale bar = 50µm; *p ≤ 0.05; **p < 0.01; ns, not significant. Data are mean +/- SEM and were compared using Mann-Whitney U test.

**Figure 2:**
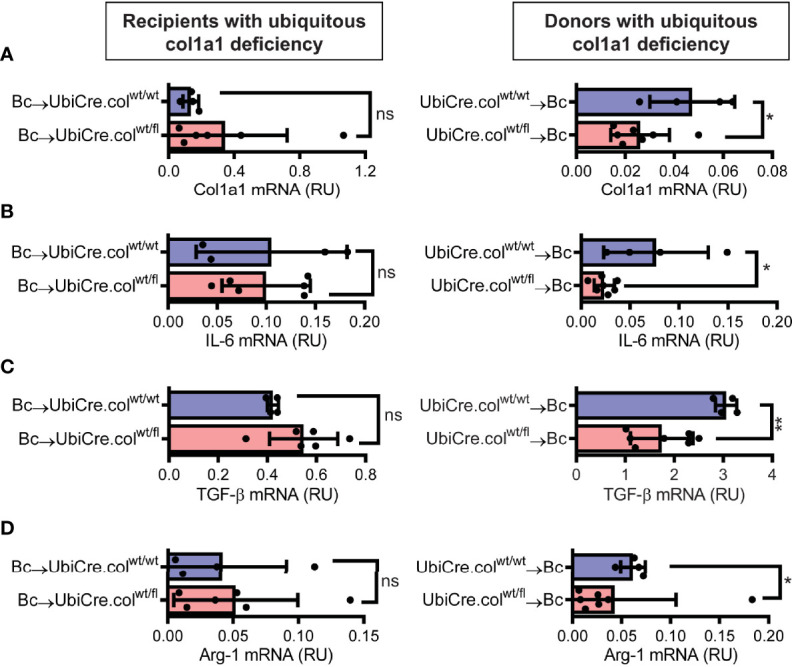
Mice with a heterozygous ubiquitous deficiency of collagen-1 used as recipients or donors of cardiac allografts - Gene expression analysis. Transplantation was performed as described in . Allografts were harvested on day 20 and gene expression was analyzed by RT-PCR. **(A–D)** Expression of col1a1, IL-6, TGF-β and Arg-1 within the allografts.*p ≤ 0.05; **p < 0.01; ns, not significant. Data are mean +/- SEM and were compared using Mann-Whitney U test.

**Figure 3:**
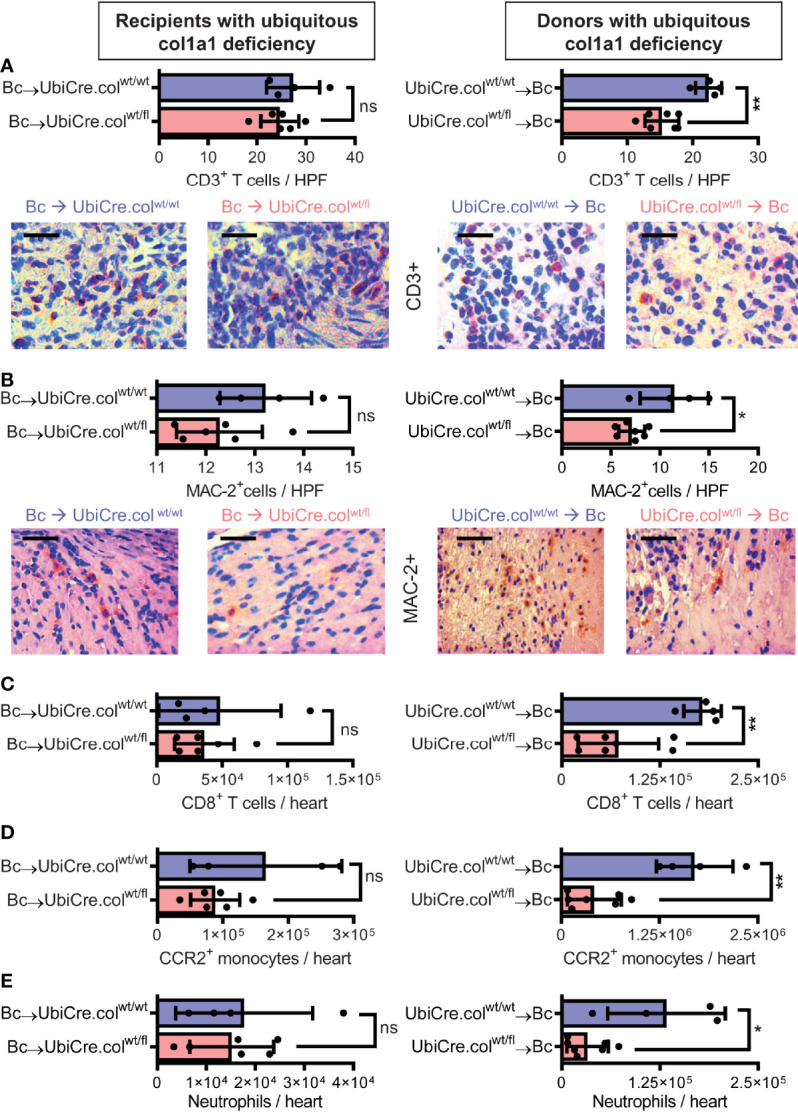
Mice with a heterozygous ubiquitous deficiency of collagen-1 used as recipients or donors of cardiac allografts - Quantification of inflammation. Transplantation was performed as described in . Allografts were harvested on day 20. **(A, B)** Quantification of allograft-infiltrating CD3^+^ T cells and MAC-2^+^ cells by immunohistology. Representative images show CD3 and MAC-2 staining in brown. **(C–E)** Quantification of allograft-infiltrating CD8^+^ T cells, CCR2^+^ monocytes and neutrophils by flow cytometry. Scale bar = 50µm; *p ≤ 0.05; **p < 0.01; ns, not significant. Data are mean +/- SEM and were compared using Mann-Whitney U test.

To determine whether cardiac allograft fibrosis is caused by donor-derived resident cells or by infiltrating cells from the recipient, we used UbiCre.col^wt/fl^ mice either as heart allograft recipients (Bc→UbiCre.col^wt/fl^) or donors (UbiCre.col^wt/fl^→Bc); as controls, we used UbiCre.col^wt/wt^ as recipients or donors, respectively. Recipients were depleted of CD4^+^ T cells and allografts analyzed on day 20 after heart transplantation for signs of chronic rejection. Histological analysis of transplants showed a significant reduction in collagen-1 deposition, fibrotic area and fibronectin expression in UbiCre.col^wt/fl^ allografts versus UbiCre.col^wt/fl^ recipients. Therefore, donor tissue-resident cells, but not recipient-derived infiltrating cells, are responsible for production of collagen-1 in allografts (**Figures 1A–C**). Expression of alpha-SMA was unchanged in both experimental combinations, indicating that the development of alpha-SMA^+^ myofibroblasts is not influenced by depletion of collagen-1 **(** **Supplementary Figures 2A, B** **).** In accordance with histological findings, quantification of mRNA revealed reduced expression of collagen-1 in UbiCre.col^wt/fl^ allografts, but not in UbiCre.col^wt/fl^ recipients **(** **Figure 2A** **)**. Also the expression of profibrotic pathways (arginase-1, Arg-1) and proinflammatory/profibrotic cytokines (IL-6, TGF-β) were reduced on an mRNA level **(** **Figures 2B–D** **).** Interestingly, the heterozygous-knock-out of collagen-1 in donor hearts also resulted in reduced inflammation in the allografts. In UbiCre.col^wt/fl^ allografts, but not in UbiCre.col^wt/fl^ recipients, we found a reduced infiltration of CD3^+^ T cells and MAC-2^+^ monocytes/macrophages by histology and a reduced infiltration of CD8^+^ T cells, CCR2^+^ monocytes and neutrophils by flow cytometry **(** **Figure 3** **)**.

### Donor-Derived Resident Hematopoietic Cells Directly Contribute to Collagen-1 Production in Allografts

**Figure 4:**
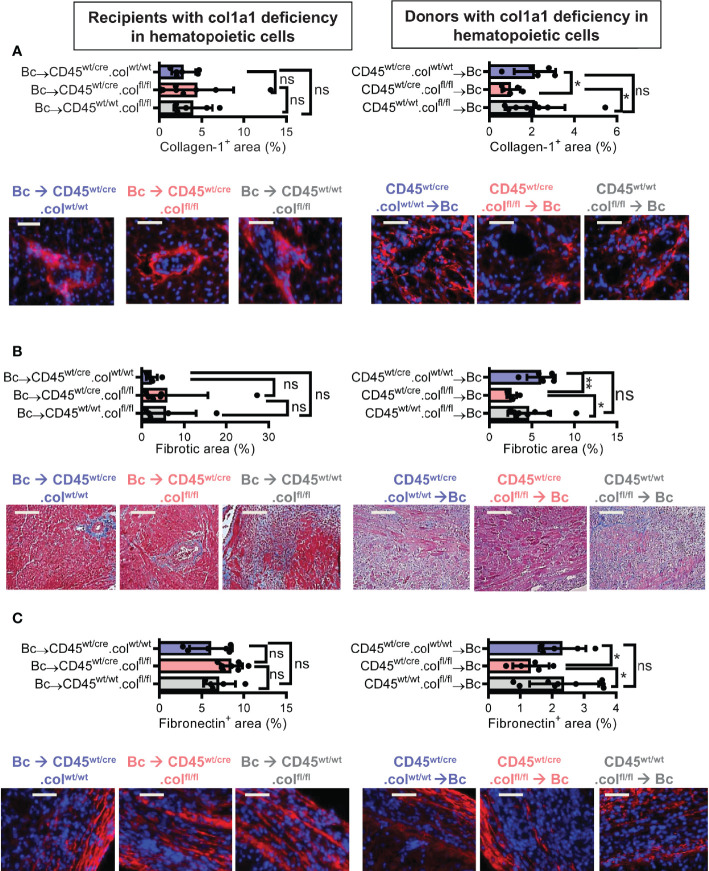
Mice with a selective deficiency of collagen-1 in hematopoietic cells used as recipients or donors of cardiac allografts – Quantification of fibrosis. C57BL/6 mice with a selective homozygous deficiency of collagen-1 (col1) in hematopoietic cells (CD45^wt/cre^.col^fl/fl^) or appropriate controls (B6.CD45^wt/cre.^col1^wt/wt^ and B6.CD45^wt/wt^.col1^fl/fl^) were used either as recipients or donors of cardiac allografts. Recipients were depleted of CD4^+^ T cells and allografts analyzed at day 20. **(A)** Collagen-1 positive (collagen-1^+^) area (%) and representative images (collagen-1 in red). **(B)** Fibrotic area (%) and representative Masson–Trichrome stainings (fibrosis in blue). **(C)** Fibronectin positive (fibronectin^+^) area (%) and representative images (fibronectin in red). Scale bar = 50µm; *p ≤ 0.05; **p < 0.01; ns, not significant. Data are mean +/- SEM and were compared using Mann-Whitney U test.

**Figure 5:**
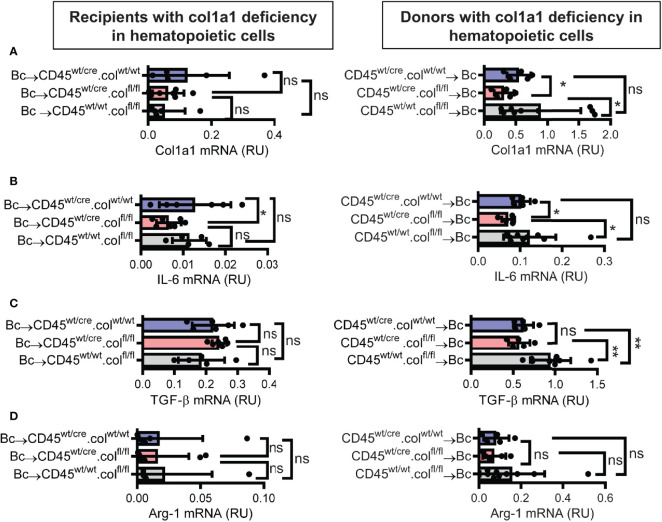
Mice with a selective deficiency of collagen-1 in hematopoietic cells used as recipients or donors of cardiac allografts – Gene expression analysis. Transplantation was performed as described in . Allografts were harvested on day 20. **(A–D)** Expression of col1a1, IL-6, TGF-β and Arg-1 within the allografts. *p ≤ 0.05; **p < 0.01; ns, not significant. Data are mean +/- SEM and were compared using Mann-Whitney U test.

Knowing that tissue resident donor-derived cells are mainly responsible for heart transplant fibrosis, we wanted to further identify the cellular source of collagen-1-producing cells. Previously, we have shown that cells of hematopoietic origin significantly contribute to fibrosis in a mouse model of unilateral ureteral obstruction and adenine nephropathy (6). We now aimed to investigate the role of hematopoietic cells as direct producers of collagen-1 in allografts. For this purpose we used C57BL/6 mice with deficiency of collagen-1 only in hematopoietic cells (CD45^wt/cre^.col^fl/fl^) and the appropriate controls (CD45^wt/cre^.col^wt/wt^ and CD45^wt/wt^.col^fl/fl^), either as donors or as recipients of cardiac allografts **(** **Figures 4**, **5** and **Supplementary Figures 3, 4** **)**. CD4^+^ T cells were depleted from recipients. Allograft fibrosis was analyzed at day 20 post-transplantation. Transplantation of donor hearts from mice with deficiency of collagen-1 only in hematopoietic cells (CD45^wt/cre^.col^fl/fl^→Bc) significantly reduced the deposition of collagen-1, overall fibrotic area and fibronectin^+^ area, whereas deficiency of collagen-1 in hematopoietic cells of the recipient had no impact on fibrosis development **(** **Figures 4A–C** **)**. These data suggest that donor-derived tissue resident hematopoietic cells in the allografts significantly contribute to allograft fibrosis whereas infiltrating hematopoietic cells of the recipient do not directly produce collagen-1. Expression of alpha-SMA was unchanged, as observed before **(** **Supplementary Figures 3A, B** **).** RT-PCR analysis of allografts confirmed the reduced expression of collagen-1 in allografts with a selective defect of collagen-1 in hematopoietic cells **(** **Figure 5A** **)**. Reduced allograft fibrosis was again associated with reduced infiltration of CD3+ T cells, CCR2+ monocytes and CD19+ B cells **(** **Supplementary Figures 4** **)** and reduced IL-6 expression **(** **Figure 5B** **)**. Expression of TGF-β and Agr-1 **(** **Figures 5C, D** **)** was not significantly altered.

**Figure 6:**
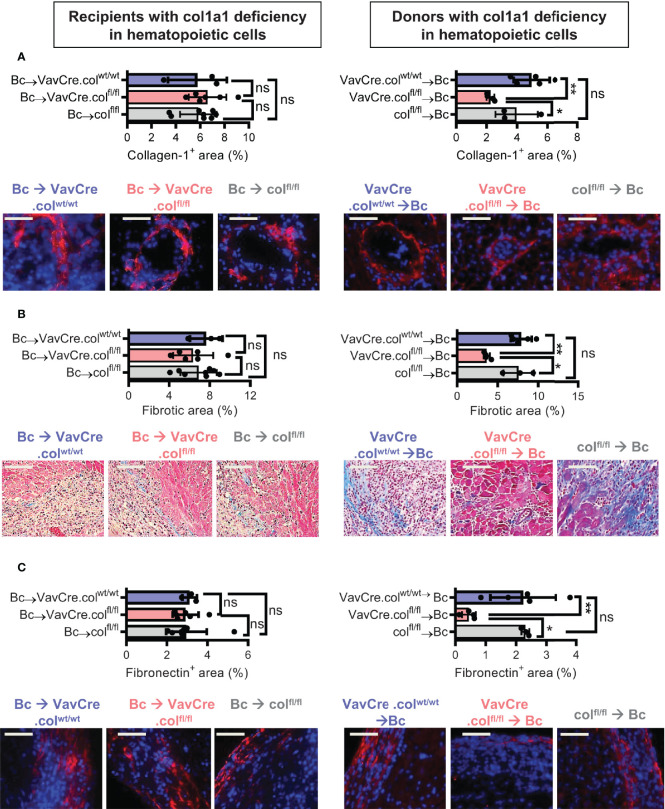
Mice with a selective deficiency of collagen-1 in hematopoietic cells used as recipients or donors of cardiac allografts – Quantification of fibrosis. C57BL/6 mice with a selective homozygous deficiency of collagen-1 in hematopoietic cells (VavCre.col^fl/fl^) or appropriate controls (VavCre.col^wt/wt^ and col ^fl/fl^) were used as either recipients or donors of cardiac allografts. Recipient were depleted of CD4^+^ T cells and allografts analyzed on day 20. **(A)** Collagen-1 positive (collagen-1^+^) area (%) and representative images (collagen-1 in red). **(B)** Fibrotic area (%) and representative Masson–Trichrome stainings (fibrosis in blue). **(C)** Fibronectin positive (fibronectin^+^) area (%) and representative images (fibronectin in red). Scale bar = 50µm; *p ≤ 0.05; **p < 0.01; ns, not significant. Data are mean +/- SEM and were compared using Mann-Whitney U test.

**Figure 7:**
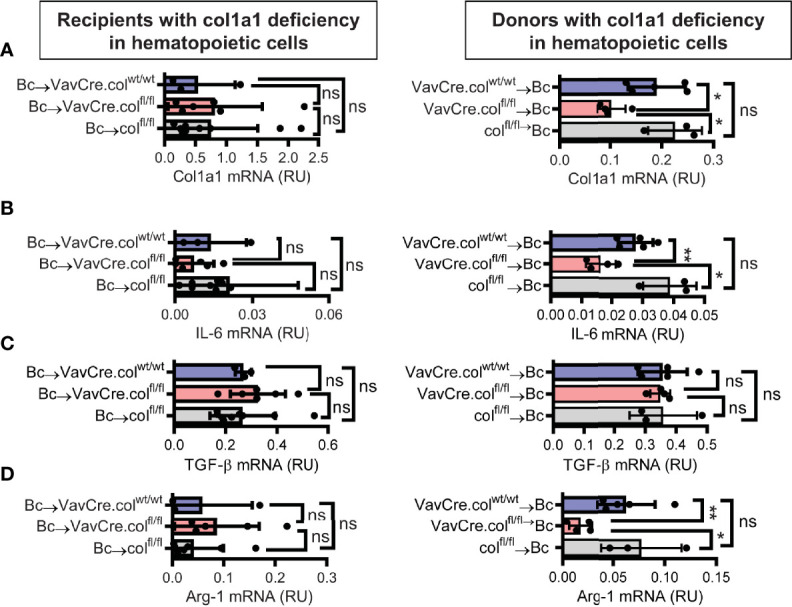
Mice with a selective deficiency of collagen-1 in hematopoietic cells used as recipients or donors of cardiac allografts – Gene expression analysis. Heart transplantation was performed as described in . Allografts were harvested on day 20. **(A-D)** Expression of col1a1, IL-6, TGF-β and Arg-1 within the allografts. *p ≤ 0.05; **p < 0.01; ns, not significant. Data are mean +/- SEM and were compared using Mann-Whitney U test.

To confirm that tissue-resident cells of hematopoietic origin markedly contribute to allograft fibrosis, we specifically deleted collagen-1 in hematopoietic cells with C57BL/6 Vav1-Cre transgenic mice as Cre-deleters (VavCre.col^fl/fl^) and used these mice either as transplant donors or recipients. Appropriate controls were included (VavCre.col^wt/wt^ and col^fl/fl^). Vav1-Cre (VavCre) transgenic mice are frequently used to specifically inactivate *floxed* genes in hematopoietic cells and their progenitors (16). Use of VavCre.col^fl/fl^ mice as heart donors prevented allograft fibrosis, as shown by significantly less collagen-1 deposition and smaller fibrotic and fibronectin^+^ areas **(** **Figures 6A–C**). Expression of collagen-1, IL-6 and Arg-1 was also significantly reduced on an mRNA level, while TGF-β remained unchanged **(** **Figures 7A–D** **)**. In contrast, use of VavCre.col^fl/fl^ mice as transplant recipients did not reduce development of allograft fibrosis for any of the analyzed parameters **(** **Figures 6**, **7** **)**. These data confirm that hematopoietic tissue-resident cells in the donor hearts are an important contributor to allograft fibrosis in this model.

### Donor-Derived Resident Macrophages Protect Against Allograft Fibrosis

Finally, we were interested in determining whether donor-derived resident macrophages were responsible for production of collagen-1 in the allografts. This hypothesis was based on our findings that less allograft fibrosis is associated with lower numbers of infiltrating macrophages (MAC-2 expressing cells) (see **Figure 3B**). MAC-2 is a galectin (β-galactoside-binding lectin of ~30 kDa) highly expressed and secreted by macrophages, known to be involved in inflammation and fibrosis (17–20). From another perspective, macrophages (mostly M2 alternative macrophages) can critically impact chronic allograft rejection (21–23). Therefore, to explore the resident macrophage contribution to heart allograft fibrosis, we depleted macrophages from BALB/c donors (including donor hearts) by repeated injection of anti-CSF1R mAb (16). CSF1 (colony stimulating factor 1) is a cytokine controlling the proliferation, differentiation, maturation and survival of cells of the mononuclear phagocyte system (10, 24, 25). Prolonged administration of blocking mAb against the CSF1receptor (anti-CSF1R) gradually eliminates resident tissue macrophages from many organs (26, 27). Treatment of BALB/c donor mice with 200 µg anti-CSF1R antibody over 3 weeks depleted CD11^+^MHC-II^+^ or F4/80^+^ macrophages with various efficacy from different organs, like heart, kidney, liver, lung or spleen **(** **Supplementary Figures 5A, B** **)**. In the heart, about 75% of tissue resident macrophages were depleted. Unexpectedly, transplantation of macrophage-depleted donor hearts resulted in more fibrosis compared to non-depleted donor hearts. This was evident by more collagen-1 deposition, more αSMA^+^ cells and larger areas with positive staining for fibronectin or matrix proteins (**Supplementary Figures 5C–F**). RT-PCR confirmed this result and showed a higher expression of collagen-1 and αSMA **(** **Supplementary Figures 6A, B** **)**. IL-6, TGFβ, Arg-1 were not significantly affected **(** **Supplementary Figures 6C–E** **)**. Our results indicate that depletion of donor organ macrophages aggravates development of chronic allograft fibrosis, suggesting a possible beneficial role for donor-resident macrophages.

## Discussion

Fibrosis is a prominent feature of chronic graft rejection and an important cause of long-term graft failure. Allograft fibrosis is considered a key target for new therapies, like depletion of fibroblasts with engineered T cells or inhibition of myofibroblast activation by various approaches (22, 28). To get deeper insight into the mechanisms of allograft fibrosis in the present study, we analyzed whether tissue-resident donor-derived cells or infiltrating cells of the recipient are responsible for fibrosis. Genetic mouse models knocking out collagen-1 served to pinpoint the source of fibrosis-promoting cells to the donor or recipient. Our experimental data formally demonstrate that tissue-resident cells from donor hearts, but not infiltrating recipient cells, are responsible for production of collagen-1 in an allograft fibrosis model. These data directly refute the idea that recipient-derived cells migrating into an allograft are responsible for fibrosis (7, 8). Our finding opens the way to novel treatment strategies that target donor cells responsible for the development of fibrosis in allografts.

In models of unilateral ureteral obstruction and adenine nephropathy, we and others have previously shown that infiltrating hematopoietic cells directly produce collagen-1 and significantly contribute to renal fibrosis (6, 29–31). However, these models differ in two important aspects from chronic allograft rejection. First, the renal models are highly inflammatory, with a pronounced non-allospecific infiltration of monocytes and T cells (6, 32, 33). Second, we have shown that blood-derived monocytes infiltrating the kidney require the presence of CD4^+^ T cells to differentiate into collagen-producing fibrocytes within the kidney (30). Depletion of CD4^+^ T cells in the cardiac allograft model could explain why peripheral blood monocytes infiltrating the allografts do not differentiate into collagen-producing cells and do not contribute to fibrosis in this experimental setup.

Depletion of CD4^+^ T cells in the recipients does not exclude that donor-derived tissue resident cells of hematopoietic origin contribute to allograft fibrosis. These tissue resident hematopoietic cells developed in the donors in the presence of CD4^+^ T cells. Their differentiation towards fibrocytes and their ability to produce collagen could persist after transplantation even in the absence of CD4^+^ T cells. To test this, we induced a cell type specific deletion of collagen-1 only in hematopoietic cells using CD45-Cre-knock-in and Vav1-Cre-transgenic deleter mice and used these mice as graft donors or recipients. As expected, deletion of collagen-1 in hematopoietic cells of the recipient had no impact on development of fibrosis. In contrast, deletion of collagen-1 in hematopoietic cells of the donor markedly reduced development of allograft fibrosis. Deletion of collagen with Vav1-Cre was somewhat more efficient that deletion with CD45-Cre, perhaps because Vav1-Cre is active in both hematopoietic cells and their progenitors (6, 16). We can therefore conclude that donor-derived hematopoietic cells contribute not only indirectly to fibrosis by the release of profibrotic factors, but also directly by production of collagen 1.

To further identify the tissue-resident collagen-producing hematopoietic cells in allografts, we depleted CSF1-dependent macrophages from donor hearts. Repeated injection of a blocking anti-CSF1R antibody into donor mice markedly reduced the number of tissue-resident macrophages in the explanted and subsequently transplanted hearts. Depletion of these donor-derived tissue-resident macrophages resulted in aggravation of allograft fibrosis, arguing against relevant collagen-production by these cells and rather indicating a potential beneficial role. Macrophages are a heterogeneous population of cells that can be involved in resolution of fibrosis and tissue remodeling (34–36). They also maintain tissue homeostasis by phagocytozing necrotic cells and inhibiting inflammatory responses including T cell activation and proliferation in different organs (37–40).

We also observed a reduced infiltration of T cells, monocytes and B cells into grafts derived from heterozygous collagen-1 deficient donors, versus appropriate control donors. These data suggest that fibrosis could induce or aggravate inflammation. Fibrosis could act as a mesh for leukocytes since extracellular matrix proteins interact with integrins on leukocytes (4, 41, 42). Increased inflammation may further aggravate fibrosis and induce a vicious cycle. In a previous study we found that IL-6 and the T cell-derived cytokine IL-3 are major drivers of cardiac allograft fibrosis (12). In our current study, the lower inflammation and the reduced expression of IL-6 in heterozygous collagen-1 deficient grafts may explain why expression of profibrotic TGF-β and arginase 1 are reduced, along with collagen-1 and other matrix proteins. More work studying the interaction of fibrosis and inflammation in allografts is needed.

In summary our data show that donor-derived cells including donor-derived hematopoietic cells are mainly responsible for allograft fibrosis in the absence of CD4^+^ T cells. Donor-derived CSF1R-dependent macrophages however, are protective and a basal production of collagen-1 by donor cells shows beneficial effects. Our results suggest that targeting the vicious cycle of fibrosis and inflammation has scientific merit as an approach to interfere with undue matrix production in allografts.

## Data Availability Statement

The raw data supporting the conclusions of this article will be made available by the authors, without undue reservation.

## Ethics Statement

The animal study was reviewed and approved by Regierung von Unterfranken (Az. 2532–2-563-17).

## Author Contributions

MM designed the study. SBa carried out most of the experimental work with assistance from SBu, FW, KS, SN, YT, and KR. SBa, EG, and MM wrote the manuscript with contributions from all authors. All authors contributed to the article and approved the submitted version.

## Funding

This work was supported by a grant from the Deutsche Forschungsgemeinschaft (MA2198/9-1).

## Conflict of Interest

The authors declare that the research was conducted in the absence of any commercial or financial relationships that could be construed as a potential conflict of interest.

## Publisher’s Note

All claims expressed in this article are solely those of the authors and do not necessarily represent those of their affiliated organizations, or those of the publisher, the editors and the reviewers. Any product that may be evaluated in this article, or claim that may be made by its manufacturer, is not guaranteed or endorsed by the publisher.

## Abbreviations

αSMA, alpha-smooth muscle actin; Arg-1, Arginase-1, Bc, BALB/c; B6, C57BL/6; CCR2, C-C chemokine receptor type 2; CD, Cluster of differentiation; Col1a1, Collagen, type I, alpha 1; CSF1R, Colony stimulating factor 1 receptor; ECM, Extracellular matrix; fl, flox; HPF, High-power field; HTx, Heart transplantation; IL, Interleukin; mAb, monoclonal antibody; M-CSF, Macrophage colony-stimulating factor; MHC, Major histocompatibility complex; TGF-β; Transforming growth factor beta; Ubi, ubiquitous; wt, wildtype.
