# Supplementary material for: Donor-But Not Recipient-Derived Cells Produce Collagen-1 in Chronically Rejected Cardiac Allografts
Source: Front Immunol. 2022 Jan 19;12:816509. doi: 10.3389/fimmu.2021.816509 (PMC8807636; doi:10.3389/fimmu.2021.816509)

Supplementary Figures Legends

Supplementary figure 1. Summary of heart transplantations

Models of heart transplantation (HTx) used in the study. C57BL/6 mice with a constitutive ubiquitous heterozygous deficiency of col1a1 (UbiCre.col$^{wt/fl}$), with a homozygous deficiency of col1a1 in hematopoietic cells (CD45$^{wt/cre}$.col$^{fl/fl}$ or VavCre.col$^{fl/fl}$) and their appropriate controls were used either as donors or recipients of cardiac allografts.

Supplementary figure 2. Mice with a heterozygous ubiquitous deficiency of collagen-1 used as recipients or donors of cardiac allografts - αSMA expression

Heart transplantation was performed as described in figure 1 and allografts were harvested on day 20. (**A)** Expression of αSMA was analyzed by immunohistology. Representative images with αSMA in red. (**B)** Gene expression of αSMA was analyzed by RT-PCR. Scale bar = 50µm; ns, not significant. Data are mean +/- SEM and were compared using Mann-Whitney U test.

Supplementary figure 3. Mice with a selective deficiency of collagen-1 in hematopoietic cells used as recipients or donors of cardiac allografts – αSMA expression

Transplantation was performed as described in figure 4 and allografts were harvested on day 20 . (**A)** Expression of αSMA was analyzed by immunohistology. Representative images with αSMA in red. One sample is missing because not enough tissue was left for αSMA detection. (**B)** Gene expression of αSMA was analyzed by RT-PCR. Scale bar = 50µm; ns, not significant. Data are mean +/- SEM and were compared using Mann-Whitney U test.

Supplementary figure 4. Mice with a selective deficiency of collagen-1 in hematopoietic cells used as recipients or donors of cardiac allografts – Quantification of inflammation

Transplantation was performed as described in figure 4. Allografts were harvested on day 20. (**A)** Quantification of allograft-infiltrating CD3$^+$ T cells by immunohistology. Representative images with CD3 staining in brown. (**B, C)** Quantification of allograft-infiltrating CCR2$^+$ monocytes and CD19$^+$ B cells by flow cytometry. Scale bar = 50µm; * $p \leq 0.05$; ** $p < 0.01$; *** $p < 0.001$; ns, not significant. Data are mean +/- SEM and were compared using Mann-Whitney U test.

Supplementary figure 5. Impact of CSF1-dependent intra-graft macrophages on allograft fibrosis

BALB/c donor mice were depleted of CSF1-dependent macrophages by treatment with anti-mouse CSF1R antibody (CD115) or an appropriate isotype control antibody (rat IgG2a) for three weeks. (**A, B)** Numbers of tissue-resident macrophages ($CD11b^+$ $MHC\text{-}II^+$ or $F4/80^+$) in various organs was quantified by flow cytometry. Cell numbers in control treated animals was set at 100% and cell numbers of anti-CSF1R treated animals is given as percent of controls. (**C-F)** Macrophage-depleted Bc donor hearts or control donor hearts were transplanted into C57BL/6 recipients. Recipients were depleted of $CD4^+$ T cells and allografts analyzed at day 20 . (**C-E)** Positive area (%) for collagen-1, αSMA and fibronectin with representative stainings in red. (**F)** Fibrotic area (%) and representative Masson–Trichrome staining of fibrosis in blue. Scale bar = 50µm; * $p \leq 0.05$. Data are mean +/- SEM and were compared using Mann-Whitney U test.

Supplementary figure 6. Impact of CSF1-dependent intra-graft macrophages on allograft fibrosis and gene expression

Depletion of intra-graft macrophages in donors and transplantation were performed as described in Suppl. Fig. 5. Allografts were harvested on day 20 and gene expression was analyzed by RT-PCR. (**A-E)** Expression of col1a1, αSMA, IL-6, TGF-β and Arg-1 within the allografts. * $p \leq 0.05$; ns, not significant. Data are mean +/- SEM and were compared using Mann-Whitney U test.

Supplemental Table: Overview of the primer sequences and primer assays for real-time PCR.

Primer	Sequence	Size
Mouse collagen (procollagen 1a) sense	5'-TGTTCAGCTTTGTGGACCTC-3'	150 bp
Mouse collagen (procollagen 1a) anti-sense	5'-TCAAGCATACCTCGGGTTTC-3'	
Mouse fibronectin sense	5'-TCCAGCCCCACCCTACAAGT-3'	282 bp
Mouse fibronectin anti-sense	5'-CCAGACCAAACCATAAGAAC-3'	
Mouse TGF-β1 sense	5'-AGCCCTGGATACCAACTATTGC-3'	92 bp
Mouse TGF-β1 anti-sense	5'-TCCAACCCAGGTCCTTCCTAA-3'	
Mouse IL-6 sense	5'-CCGGAGAGGAGACTTCACAG-3'	134 bp

Mouse IL-6 anti-sense	5'-CAGAATTGCCATTGCACAAC-3'	
Mouse Arg-1 sense	5'-CTCCAAGCCAAAGTCCTTAGAG-3'	185 bp
Mouse Arg-1 anti-sense	5'-AGGAGCTGTCATTAGGGACATC-3'	

Primer	Assay name	Locus
Mouse hypoxanthine guanine phosphoribosyl transferase (Hprt)	Mm_Hprt_1_SG	NM_013556
Mouse β-2 microglobulin (B2m)	Mm_B2m_2_SG	NM_009735
Mouse glyceraldehyde-3-phosphate dehydrogenase (Gapdh)	Mm_Gapdh_3_SG	NM_008084
Mouse alpha-smooth-muscle-actin (α-SMA)	Mm_Acta2_1_SG	NM_007392

Supplementary Fig. 1

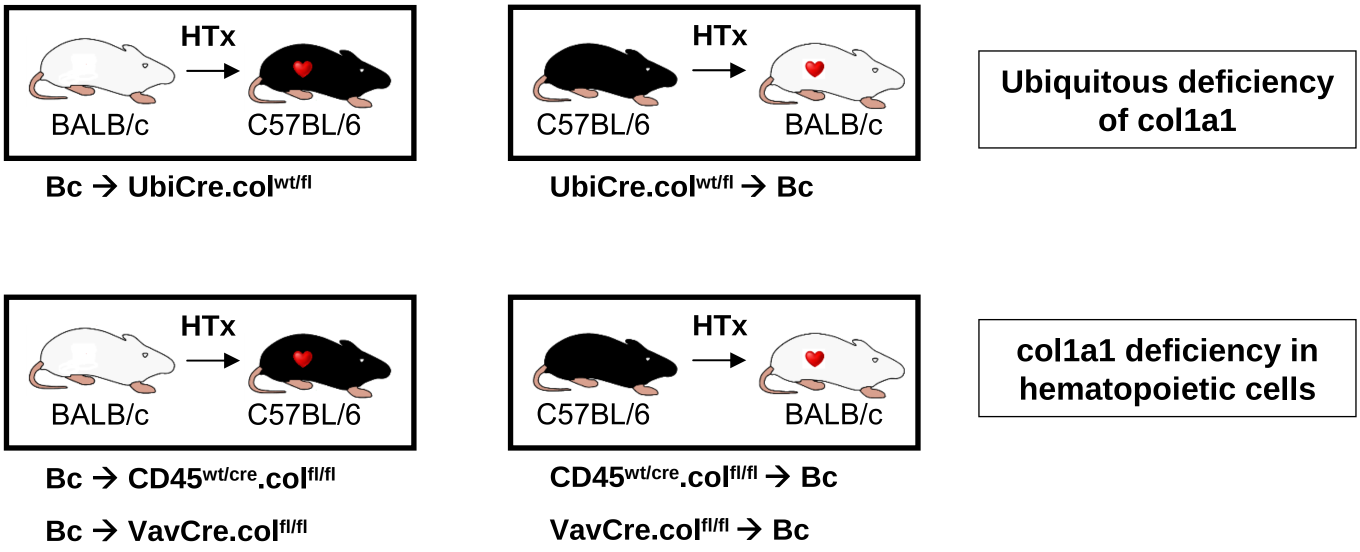

Supplementary Fig. 2

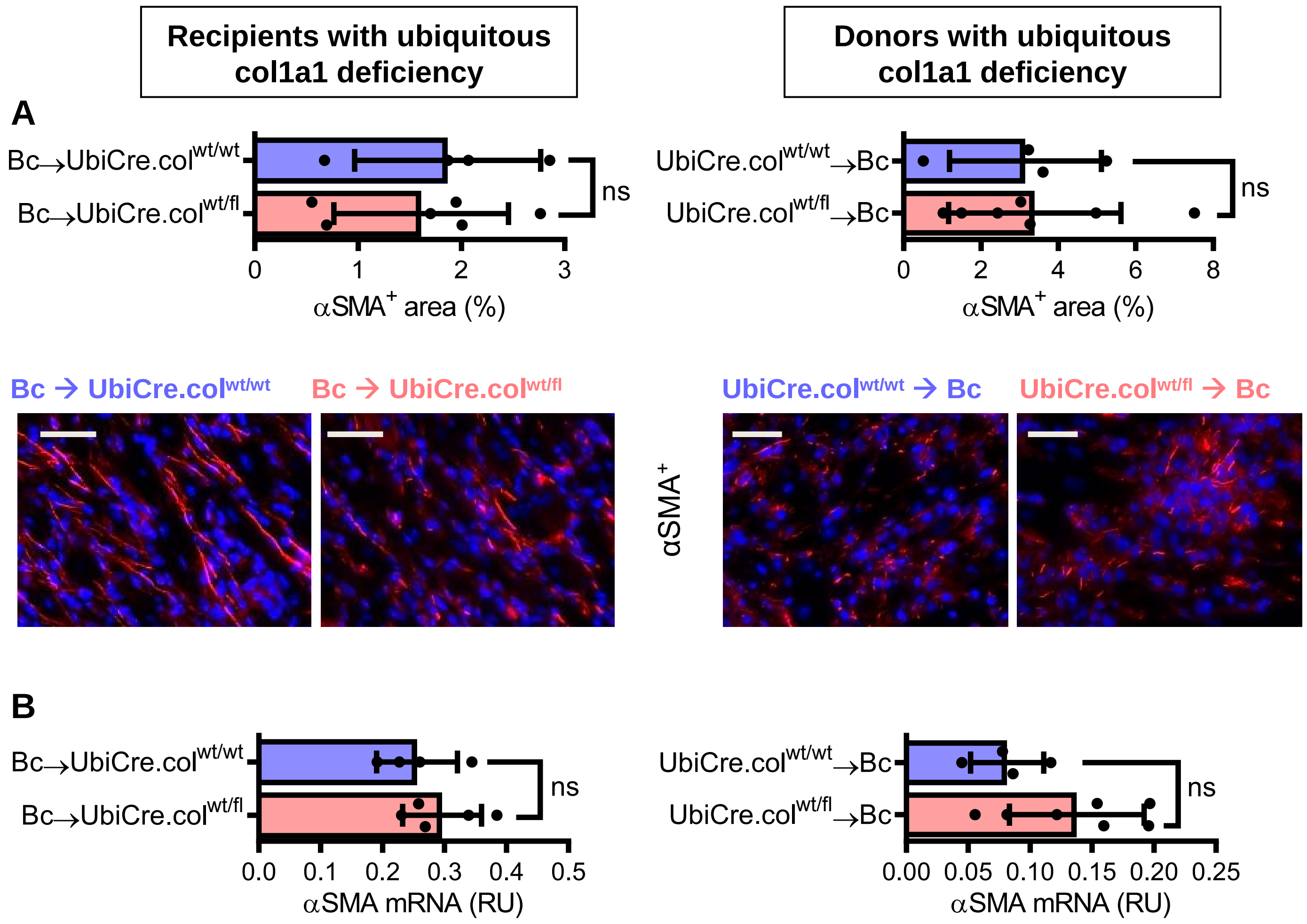

Supplementary Fig. 3

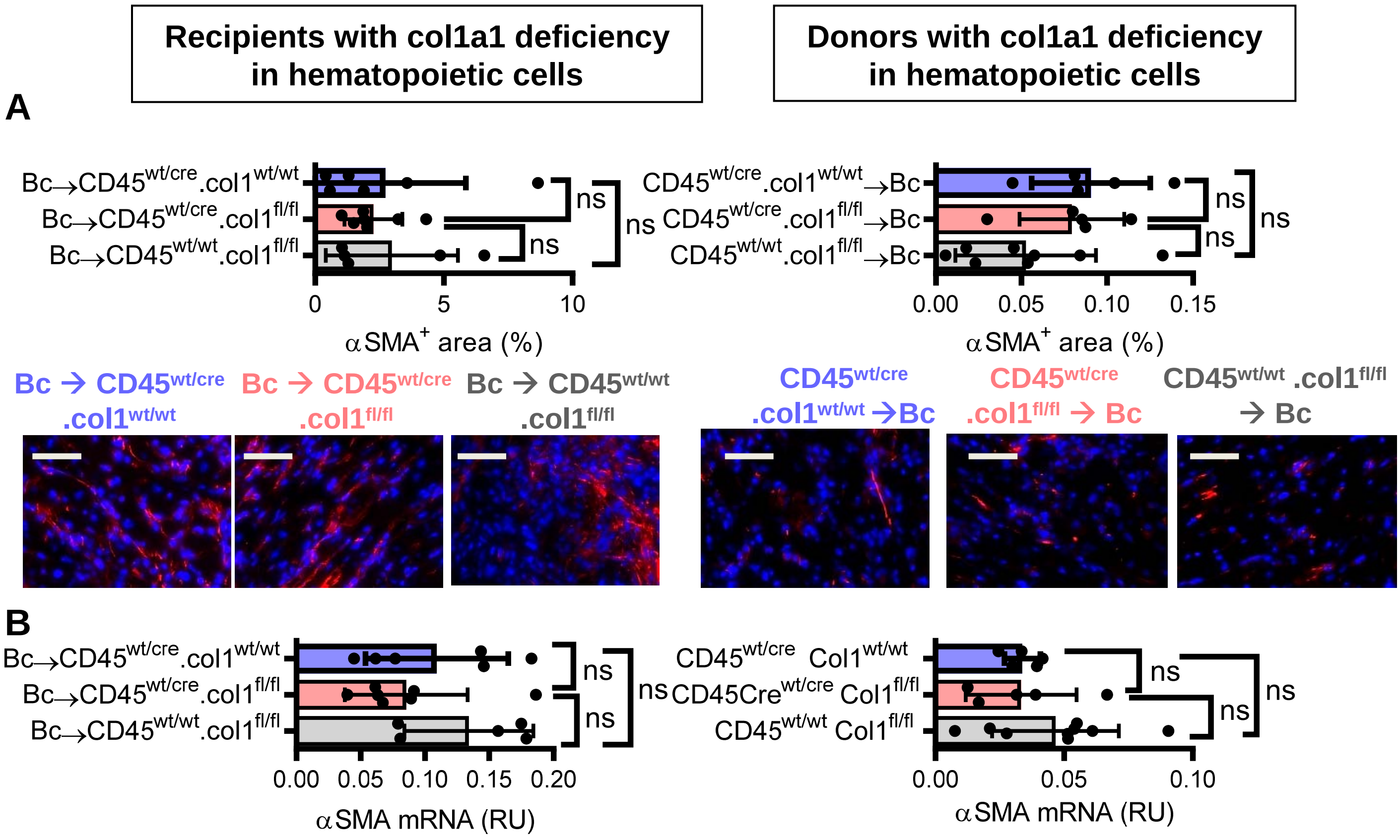

Supplementary Fig. 4

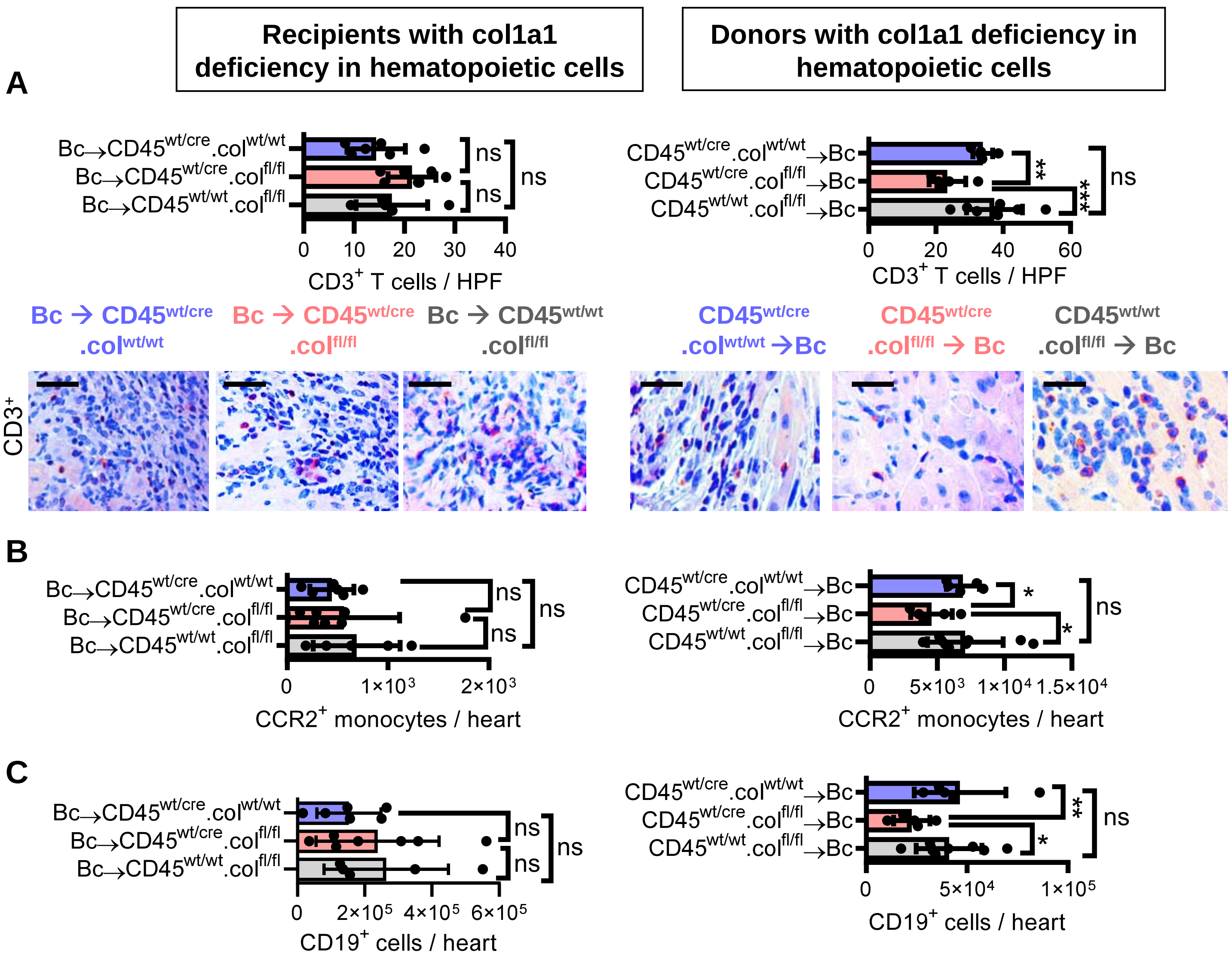

Supplementary Fig. 5

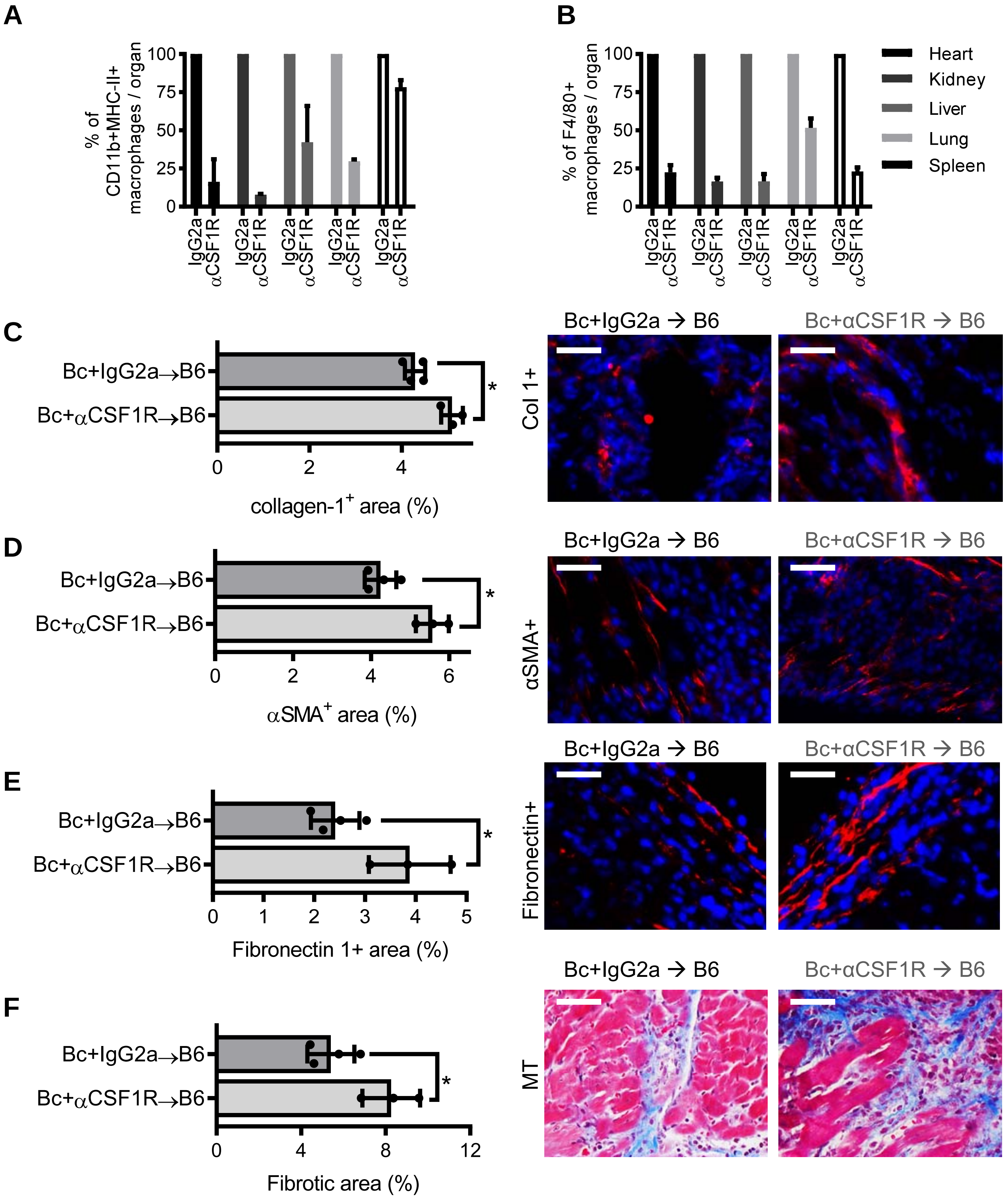

Supplementary Fig. 6

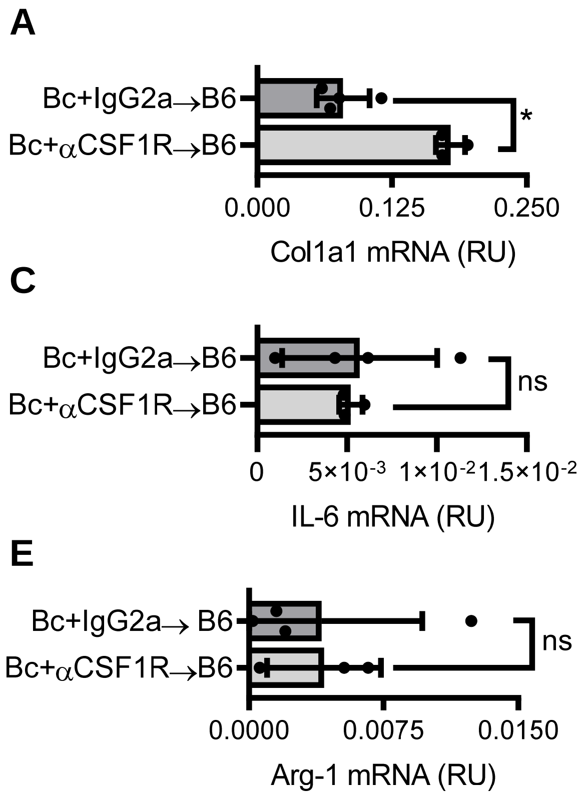

Supplement: Supplementary file 1 [file DataSheet_1.pdf]
